# The Synergistic Effects of Resveratrol combined with Resistant Training on Exercise Performance and Physiological Adaption

**DOI:** 10.3390/nu10101360

**Published:** 2018-09-22

**Authors:** Nai-Wen Kan, Mon-Chien Lee, Yu-Tang Tung, Chien-Chao Chiu, Chi-Chang Huang, Wen-Ching Huang

**Affiliations:** 1 Center for General Education, Taipei Medical University, Taipei 11031, Taiwan; kevinkan@tmu.edu.tw; 2 Graduate Institute of Sports Science, National Taiwan Sport University, Taoyuan 33301, Taiwan; 1061304@ntsu.edu.tw (M.-C.L.); chiu2295@yahoo.com.tw (C.-C.C.); 3 Graduate Institute of Metabolism and Obesity Sciences, Taipei Medical University, Taipei City 11031, Taiwan; f91625059@tmu.edu.tw; 4 Nutrition Research Center, Taipei Medical University Hospital, Taipei 11031, Taiwan; 5 Department of Exercise and Health Science, National Taipei University of Nursing and Health Sciences, Taipei 11219, Taiwan

**Keywords:** resveratrol, resistance exercise, hypertrophy, physiological adaption, performance

## Abstract

The comprehensive studies done on resveratrol (RES) support that this polyphenol has multiple bioactivities and is widely accepted for dietary supplementation. Furthermore, regular exercise is known to have benefits on health and is considered as a form of preventive medicine. Although the vast majority of prior studies emphasize the efficacy of aerobic exercise in promoting physiological adaptions, other types of exercise, such as resistance exercise and high-intensity interval training (HIIT), may achieve similar or different physiological outcomes. Few studies have looked into the effectiveness of a combinational, synergistic approach to exercise using a weight-loading ladder climbing animal platform. In this study, ICR mice were allocated randomly to the RES and training groups using a two-way ANOVA (RES × Training) design. Exercise capacities, including grip strength, aerobic performance, and anaerobic performance, were assessed and the physiological adaptions were evaluated using fatigue-associated indexes that were implemented immediately after the exercise intervention. In addition, glycogen levels, muscular characteristics, and safety issues, including body composition, histopathology, and biochemistry, were further elucidated. Synergistic effects were observed on grip strength, anaerobic capacities, and exercise lactate, with significant interaction effects. Moreover, the training or RES may have contributed significantly to elevating aerobic capacity, tissue glycogen, and muscle hypertrophy. Toxic and other deleterious effects were also considered to evaluate the safety of the intervention. Resistance exercise in combination with resveratrol supplementation may be applied in the general population to achieve better physiological benefits, promote overall health, and promote participation in regular physical activities.

## 1. Introduction

Resveratrol (trans-3,4′,5-trihydroxystilbene, RES), a stilbenoid, is a natural polyphenol that has be widely investigated for its bioactivity and potential therapeutic applications. RES occurs naturally in a wide variety of plant species, including grapes, blueberries, raspberries, and mulberries [1]. Moreover, RES is a phytoalexin, which is a class of compounds produced by many plants in response to infection by pathogens or to physical injury due to, for example, cutting, crushing, or ultraviolet radiation [2]. Both cis- and trans-resveratrol are fat-soluble and bound to a glucose molecule, also called piceid [3]. One theory to explain the “French Paradox”, a term used to describe the phenomenon of the French people having a relatively low incidence of coronary heart disease (CHD) despite their relatively high consumption of high-fat foods, focuses on the positive impact of dietary behaviors such as a high consumption of red wine and food diversity [4]. Since the confirmation of its presence in red wine in the early 1990s, the effects of resveratrol on health-related issues have been widely studied by the scientific community. Previous studies have reported on the positive effects of RES in the realms of cancer prevention [5], cardiovascular disease prevention [6], glucose homeostasis [7], neurodegenerative diseases [8], and longevity due to its anti-oxidation, anti-inflammation, and calorie restriction mimetic qualities [9]. Resistance exercise (weight training) is a form of exercise that improves muscular strength/endurance by using the practitioner’s bodyweight, weighted barbells, dumbbells and elastic bands as resistance for muscles.

Resistance exercise contributes many factors, including mechanical tension, muscle damage, and metabolic stress to mediate the hypertrophic process in exercise-induced muscle growth/hypertrophy [10]. Age-related muscle loss is an important health issue. Individuals lose an average of 3% to 8% of their muscle mass per decade, and this rate increases significantly after 70 years of age [11,12]. However, although the literature has emphasized the beneficial effects of aerobic activity, little encouragement has been offered for resistance training. Recently, resistance exercise has been shown to have positive effects on functional improvements and disease prevention, including muscle growth, resting metabolism recharging, body compositions, elderly physical function, chronic metabolic disease, and mental health [13,14]. Athletes in different activity areas were asked to incorporate resistance exercise into their regular training regimens and the before-after effects on performance and injury incidence were evaluated [15]. A position statement on resistance training in a youth population addressing technical skill and competency was developed that promoted the performance of a variety of resistance training exercises at appropriate intensities and volumes while providing youth an opportunity to participate in programs that are safe and effective [16].

A previous study by the present authors elucidated the beneficial effects of RES on aerobic-exercise-induced peripheral fatigue through improving physiological adaption and energy content [17]. Another report also demonstrated that RES, in combination with aerobic exercise, significantly elevated not only muscle hypertrophy but also muscle torque and power in an elderly population [18]. However, the dual effect of RES antioxidant supplementation in human studies is a well-known issue. Free radical production during exercise might be a necessary trigger for adaptations to exercise stimuli leading to the well-known improvements in exercise performance or exercise-induced positive effects on the metabolism. Correspondingly, reducing the increase in production of exercise-induced oxidative stress by using antioxidant supplements is now being discussed as being counterproductive and even preventing health-promoting effects of exercise [19]. Based on the above, RES is a widely accepted dietary supplement and resistance exercise is an increasingly accepted form of exercise with health-promotion and functional benefits. However, few studies have addressed the effects of RES supplementation in combination with anaerobic exercise and resistance training. Thus, the purpose of this study was to determine the effects of a 4-week resistance exercise program combined with RES on functional performance, physiological adaption, muscular hypertrophy, and safety.

## 2. Materials and Methods

### 2.1. Materials

**Figure 1:**
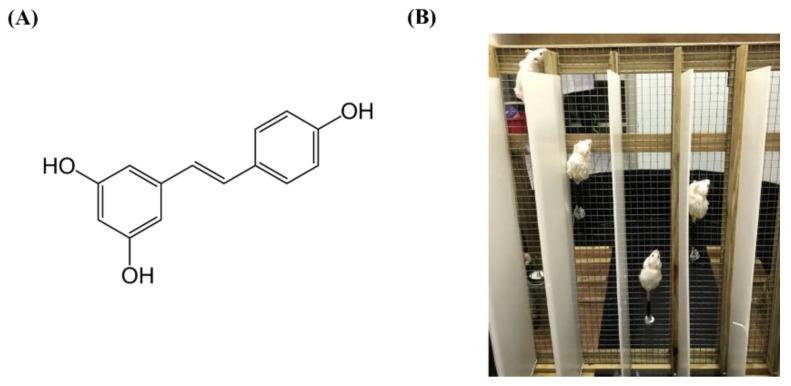
The structure of resveratrol (**A**) and the climbing device (**B**) for resistance training.

The nutraceutical ingredient trans-resveratrol (>98%) used in this study was purchased from Vitacost (Boca Raton, FL, USA), shown in Figure 1A, and the resistance exercise program used was adapted from a program used in a previous study [20]. The exercise equipment was about 100 cm in height with variable angles (30–85 degrees) to adjust the intensity of protocol. The ladder was modified to use a 1 cm^2^ stainless net in order to avoid climbing arrest if a load became stuck into the ladder gaps, shown in Figure 1B, and a screw washer was used to increase the load according to the bodyweight of each animal for appropriate adjustment of intensity.

### 2.2. Animals and Experiment Design

Male ICR mice (6 weeks old, SPF grade) from BioLASCO Taiwan (Yi-Lan, Taiwan) were used in this animal study. These mice were familiarized with the ladder equipment and acclimatized to the environment and dietary differences during the 1-week period immediately prior to the training protocol. All animals were given a standard laboratory diet #5001 (PMI Nutrition International, MO, USA) and distilled water ad libitum, and maintained in stable photoperiod, temperature, and humidity conditions (12-h light/12-h dark cycle, 24 ± 2 °C, and 55–65%, respectively) during the experiment. The Institutional Animal Care and Use Committee (IACUC) of National Taiwan Sport University inspected all of the animal experiments in this study and the study conformed to the IACUC-10604 protocol guidelines approved by the IACUC ethics committee.

**Figure 2:**
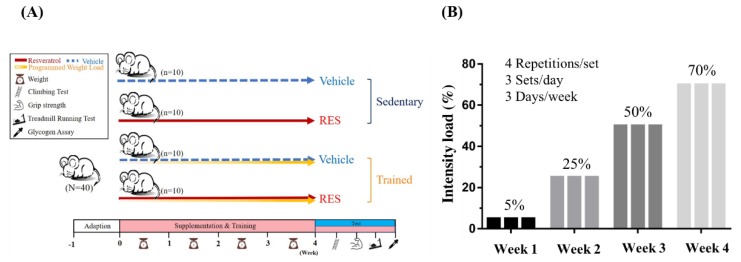
The experimental procedure to evaluate the effects of progressive resistance exercise and resveratrol on aerobic capacity, anaerobic capacity, physiological adaption, and safety (**A**). The incremental loading intensity applied to current training protocol (**B**).

The recommended resveratrol dosage of 25 mg/kg bodyweight, which was applied to our previous exercise fatigue study [17], was administrated by oral gavage for 4 consecutive weeks. The two-way experiment (Training × Resveratrol) was designed for 4 groups (*n* = 10/group) to figure out the main effects and interactions on physiological assessments. The detailed experimental procedure is illustrated in Figure 2A. The weight, dietary, and social behaviors were monitored during the supplementation period and the daily training and interventions began at a regular time. Physical fitness was assessed using direct forelimb grip strength, aerobic endurance, and anaerobic performance, including assessments of ladder-climbing time and time taken to reach exhaustion for strength and endurance parameters, respectively. The exercise-related biochemistries were immediately assessed after a fixed exercise time/intensity for physiological adaption.

### 2.3. Anaerobic Exercise Training and Capacity Test

The resistance training protocol was performed 3 days/week for 4 weeks and the indicated intensity load was adjusted by individual animal weight using the protocol, as seen in Figure 2B. In resistance training, the climbing procedures used 4 repetitions/set and 3 sets/day, with 2 min of rest provided between sets. The equipment was set into water of 5 cm in depth to provide negative stimulation in order to increase climbing motivation. Performance was evaluated as the climbing time, and the number of climbs until exhaustion was reached was used to evaluate the anaerobic performance.

### 2.4. Aerobic Exercise Endurance Performance Test

A motor-driven treadmill that was designed for rodents (model MK-680, Muromachi Kikai, Tokyo, Japan) was used to evaluate aerobic endurance and the electric shock grid was used to increase test motivation with veterinarian surveillance. All of the mice were initially acclimated to running on a motorized treadmill at 10 m/min, 5% grade, for 5 min/day during the week prior to the exhaustive exercise. The mice were run on the treadmill at an initial speed of 15 m/min and grade 15% for 2 min, and then every subsequent 2 min the speed was increased by 3 m/min until exhaustion [21]. Exhaustion was defined as the point at which mice maintained continuous contact with the shock grid for 5 s. Aerobic endurance capacity was expressed as time-to-exhaustion (min).

### 2.5. Forelimb Grip Strength

A low-force testing system (Model-RX-5, Aikoh Engineering, Nagoya, Japan) was applied to measure grip strength, as described previously [22].

### 2.6. Fatigue-Associated Biochemical Variables

The effect on fatigue-associated biochemical indexes, based on our previous reports [23], were slightly modified to accurately reflect the actual physiological status. The blood was sampled by submandibular blood collection immediately after 15 min of acute exercise with constant intensity, and was tested to measure glucose, lactate, blood urine nitrogen (BUN), creatine kinase (CK), and ammonia levels. The blood samples were centrifuged at 1000× *g* and 4 °C for 15 min after complete clotting for serum separation and analyzed using an autoanalyzer (Hitachi 7060, Hitachi, Tokyo, Japan).

### 2.7. Clinical Biochemical Profiles

All of the mice were euthanatized by 95% CO_2_ asphyxiation one hour after the last treatment and their blood was immediately sampled via cardiac puncture. Serum was separated using centrifugation, with clinical biochemical variables, including aspartate aminotransferase (AST), alanine transaminase (ALT), creatine kinase (CK), glucose (GLU), lactate dehydrogenase (LDH), blood urea nitrogen (BUN), creatinine (CREA), uric acid (UA), albumin (ALB), triglycerides (TG), and total protein (TP), measured using an autoanalyzer (Hitachi 7060).

### 2.8. Body Composition and Glycogen Content Analysis

The important visceral organs, including liver, kidney, heart, lung, muscle (gastrocnemius), MT (thigh muscle), EPF (epididymal fat pad) and BAT (brown adipocyte tissue) were accurately excised and weighed after sacrifice. Then, the organs were preserved in 10% formalin for further histopathology and immunohistochemistry procedures. Part of the muscle samples were kept in liquid nitrogen for glycogen content analysis, as described previously [24].

### 2.9. Immunohistochemical Staining

The muscle tissues (gastrocnemius) embedded in paraffin were further analyzed to study the effects of training and resveratrol supplementation on type I and type II fibers. The primary antibodies of myosin-heavy chain fast (WB-MHCf) and myosin-heavy chain slow (WB-MHCs) were purchased from Novocastra (Leica Biosystem, Wetzlar, Germany) and applied in order to distinguish the fiber types. The epitope of MHCf and MHCs was retrieved using ER2 retrieval solution (AR9640, Leica Biosystem, Wetzlar, Germany), followed by primary incubation. The detection kits (Bond Polymer Refine Detection & Bond Polymer Refine Red Detection) used an automated BondMax double staining system. Finally, the results were examined by a veterinary pathologist under a light microscope that was equipped with a CCD camera (BX-51, Olympus, Tokyo, Japan).

### 2.10. Histopathology

The visceral organs preserved in 10% formalin were trimmed to provide tissue sections of 4 μm thickness, which were then embedded in paraffin. Tissue sections were further stained with hematoxylin and eosin (H&E) and examined by a veterinary pathologist under the abovementioned CCD-camera-enabled light microscope (BX-51, Olympus, Tokyo, Japan).

### 2.11. Statistical Analysis

The data were represented as mean ± standard error of mean (SEM). Two-way analysis of variance (resistance training × resveratrol supplementation) was used to analyze the statistical differences among the groups in terms of physical activity, biochemistry, body composition, diet, and glycogen content to verify the main and interaction effects by SPSS v19.0 analysis. Eta-square (η^2^) is an effect size measurement for the analysis of variance (ANOVA). It measures the strength of the effect on a continuous field. Data were considered statistically significant when the probability of a type I error was <0.05.

## 3. Results

### 3.1. The Effects of Climb Training and Resveratrol on Grip Strength

**Figure 3:**
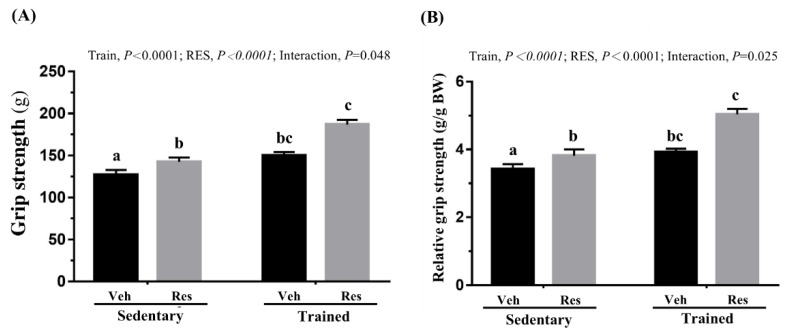
climb training (Trained) and/or resveratrol (RES) supplementation on absolute forelimb grip strength (**A**) and forelimb grip strength (%) relative to bodyweight (**B**). Data are mean ± SEM for *n* = 10 mice per group. Columns with different superscript letters (a, b, c) are significantly different at *p* < 0.05. The abbreviations Veh and RES represented the vehicle and resveratrol supplement, respectively.

In terms of forelimb absolute grip strength, as seen in Figure 3A, the climb training (Trained) + vehicle (Veh) and Trained + Res groups increased grip strength, as compared to the untrained groups, (Sedentary, Sed) + Veh and Sed + RES, with the main effect of climb training (*F* (1,36) = 41.96, *P* < 0.0001, eta = 0.538) and the effect of climb training on grip strength significantly increasing the grip strength of training groups as compared to sedentary groups. In addition, RES supplementation significantly increased the grip strength (*F* (1,36) = 25.2, *P* < 0.0001, eta = 0.412). Therefore, the Trained + RES group had significantly higher grip strength than the Sed + RES group, with a significant interaction effect (*F* (1,36) = 25.2, *P* = 0.048, eta = 0.150). A previous study found grip strength to correlate positively with anthropometric factors such as age, weight, and body mass index [25]. The similar analytic trends found in this study may further validate the results of grip strength adjusted to individual bodyweight, as seen in Figure 3B.

### 3.2. The Effects of Climb Training and Resveratrol on Anaerobic Exercise Performance

**Figure 4:**
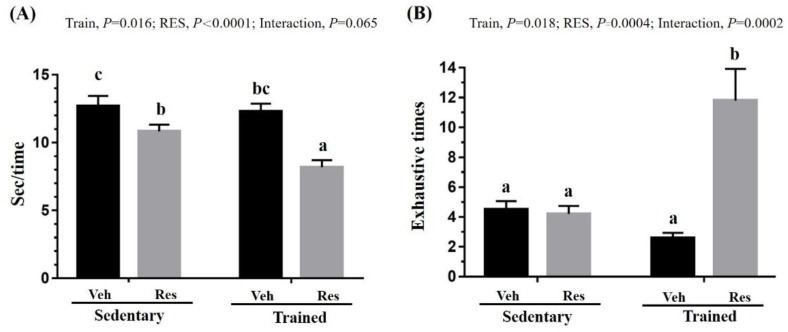
Effect of climb training (Trained) and/or resveratrol (RES) supplementation on anaerobic endurance performance, including time for each climbing (**A**) and exhaustion times for climbing (**B**). Data are mean ± SEM for *n* = 10 mice per group and the columns with different superscript letters (a, b, c) are significantly different at *P* < 0.05. The abbreviations Veh and RES represented the vehicle and resveratrol supplement, respectively.

Speed and anaerobic endurance performance were used to assess the indexes of anaerobic exercise performance. In terms of speed performance, as seen in Figure 4A, the climb training and RES supplementation significantly improved the time performance (climb training: *F* (1,36) = 6.39, *P* = 0.016, eta = 0.151; RES supplementation: *F* (1,36) = 24.85, *P* < 0.0001, eta = 0.408). However, the interaction effect did not show a significant difference (*F* (1,36) = 3.61, *P* = 0.065, eta = 0.091) even though the time performance of the Trained + RES group was significantly better than Sed + RES group (*P* < 0.05). In terms of anaerobic endurance capacity, the exhaustion times for repetitive climbing were also evaluated as shown in Figure 4B. The main effects of climb training and RES demonstrated significant differences (climb training: *F* (1,36) = 6.19, *P* = 0.018, eta = 0.147; RES supplementation: *F* (1,36) = 15.1, *P* = 0.0004, eta = 0.296). The Trained + RES group was significantly higher than the Sed + RES group, with a significant interaction effect (*F* (1,36) = 17.21, *P* = 0.0002, eta = 0.323).

### 3.3. The Effects of Climb Training and Resveratrol on Aerobic Exercise Performance

**Figure 5:**
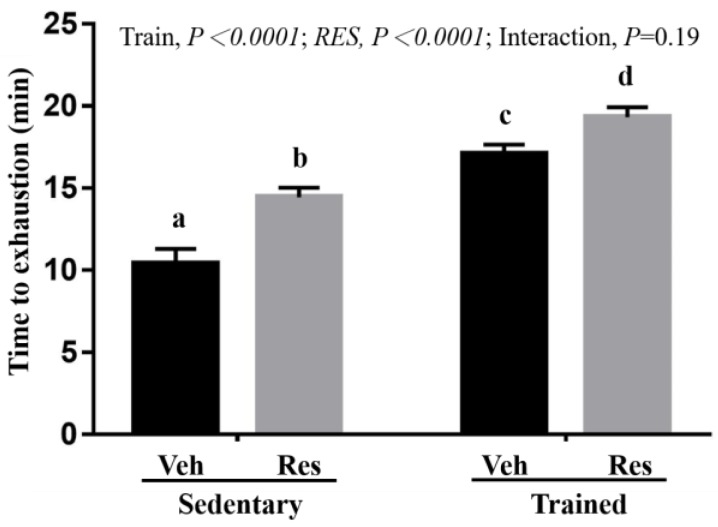
Effect of climb training (Trained) and/or resveratrol (RES) supplementation on aerobic endurance performance. Data are mean ± SEM for *n* = 10 mice per group and the columns with different superscript letters (a, b, c, d) are significantly different at *P* < 0.05. The abbreviations Veh and RES represented the vehicle and resveratrol supplement, respectively.

The treadmill test has been applied widely to assess cardiorespiratory ability (VO_2_ max) and aerobic endurance capacity. In the exhaustive running test, shown in Figure 5, a significant difference was observed for the main effects of both training and RES (*F* (1,36) = 74.03, *P* < 0.0001, eta = 0.673 and *F* (1,36) = 21.73, *P* < 0.0001, eta = 0.376, respectively). The Sed + RES group and Trained + RES group were significantly higher than the Sed + Veh group and Trained + Veh group, respectively, although the training combined with RES treatment (Trained + RES group) did not show synergistic effects due to the lack of a significant interaction effect (*F* (1,36) = 1.78, *P* = 0.19, eta = 0.047).

### 3.4. The Effects of Climb Training and Resveratrol on Fatigue-Associated Biochemistries

Table 1 shows the results of the assessment of the fatigue-associated indexes, including GLU, Lactate, BUN, CK and NH_3_, that was conducted immediately after 15 min of acute exercise. No significant differences in GLU, BUN, or CK were observed among the 4 indicated groups discounting the main and interaction effects. In terms of lactate levels, the exercise training groups had significantly lower levels than the sedentary groups, with the main effect of exercise training (*P* = 0.0035) and RES supplement identified as the significant main effect (*P* < 0.0001). The interaction effect for training and supplementation was significant after the 4-week resistance training program. In addition, ammonia levels exhibited a significant difference in the supplement’s main effect (*P* = 0.0075) but not in the main effect of training or interaction effect (*P* = 0.69 and 0.14, respectively).

### 3.5. The Effects of Climb Training and Resveratrol on Clinical Biochemistries

The serum was further analyzed at the end of the experiment for related clinical biochemistries, as seen in Table 2. No significant differences in main and interaction effects were identified on AST, TG, CK, LDH, BUN, Creatinine, ALB, TP, and GLU indexes and the indicated groups did not differ significantly. The ALT index showed significant differences in RES main effect (*P* = 0.008), with this effect significantly higher in the Sed + RES and Trained + RES groups than the Sed + Veh and Trained + Veh groups. However, the main effects and interaction effects of training were not significantly different. Besides, the UA index differed significantly not only in terms of the main effect of training but also in terms of the main effect of RES, with significantly higher effects in the Sed + RES and Trained + RES groups than the Sed + Veh and Trained + Veh groups but no significant differences in terms of the interaction effect (*P* = 0.726).

### 3.6. The Effects of Climb Training and Resveratrol on Tissue Glycogen Contents

**Figure 6:**
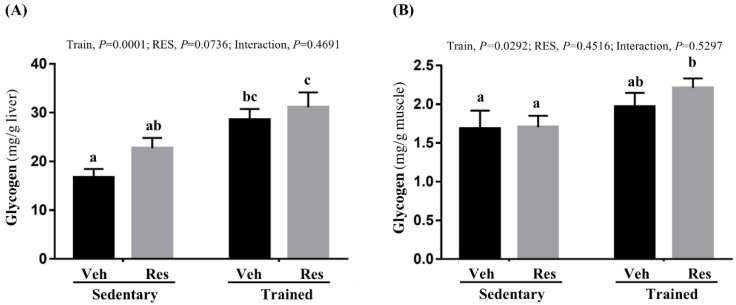
Effect of climb training (Trained) and/or resveratrol (RES) supplementation on hepatic (**A**) and muscle (**B**) glycogen level. Data are mean ± SEM for *n* = 10 mice per group. Bars with different superscript letters (a, b, c) are significantly different at *P* < 0.05. The abbreviations Veh and RES represented the vehicle and resveratrol supplement, respectively.

Glycogen is primarily stored in the liver and muscle tissues for the purpose of energy homeostasis and demands. In terms of liver glycogen content, as seen in Figure 6A, the climb training regimen increased the liver glycogen content in the trained groups to a level significantly higher than that in the untrained groups (*F* (1,36) = 19.05, *P* < 0.0001, eta = 0.346). In terms of RES effects, the main effect of RES supplementation was not significant (*F* (1,36) = 3.39, *P* = 0.074, eta = 0.086). Furthermore, the synergistic effects of training and RES supplementation was not significant due to the lack of a significant interaction effect (*F* (1,36) = 0.54, *P* = 0.47). In terms of muscular glycogen content, shown in Figure 6B, while a significant main effect was not supported for RES supplementation (*F* (1,36) = 0.58, *P* = 0.45, eta = 0.016), it was supported for the training (*F* (1,36) = 5.16, *P* = 0.029, eta = 0.125). In addition, the synergistic effects of training and RES supplement were not significant due to the lack of a significant interaction effect (*F* (1,36) = 0.4, *P* = 0.53).

### 3.7. The Effects of Climb Training and Resveratrol on Growth and Body Composition

The growth curve over the duration of climb training did not significantly differ between the groups without main and interaction effects, as seen in Table 3. In terms of dietary data, the significant main and interaction effects of training were found in food and water consumption, with the Trained + Veh and Sed + RES groups significantly higher than the Sed + Veh group in this category (*P* < 0.05). Body composition variables, including liver, muscle, heart, lung, kidney, epididymal fat pad (EFP), and BAT, did not differ significantly between the groups without significant main and interaction effects. Remarkably, the thigh muscle (MT) showed a significant training main effect (*P* = 0.035), with the training groups earning significantly higher scores than the sedentary group. The percentage of organ and tissue weight, adjusted by individual weight, also exhibited the same analytic results (data not shown).

### 3.8. The Effects of Climb Training and Resveratrol on Histological Observation

**Figure 7:**
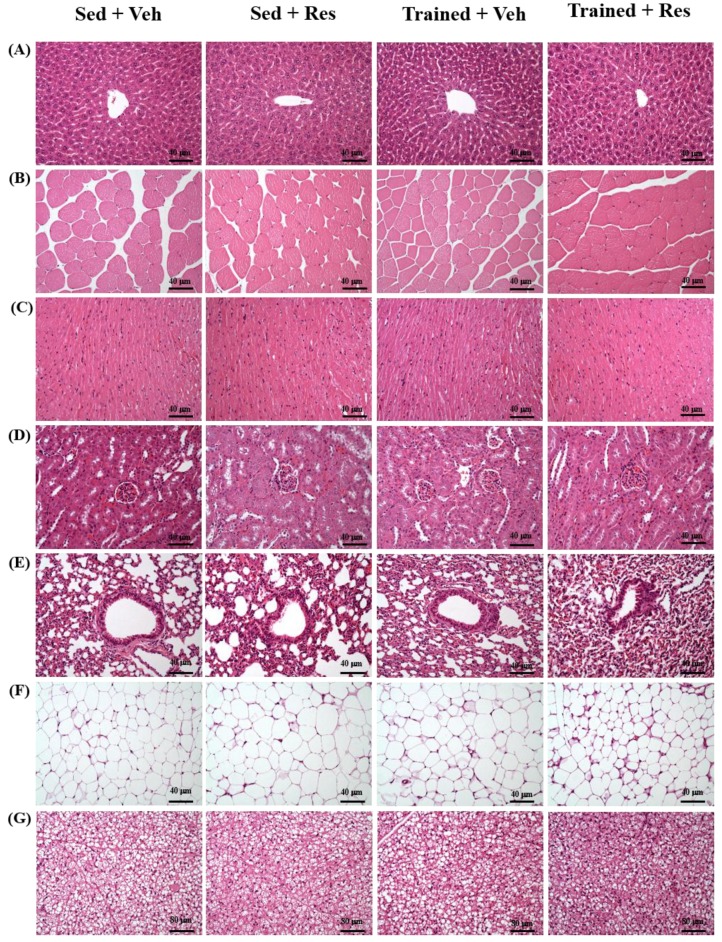
Effect of climb training (Trained) and/or resveratrol (RES) supplementation on the morphology of (**A**) liver; (**B**) skeletal muscle; (**C**) heart; (**D**) kidney; and (**E**) lung (**F**) white adipose tissue (WAT) (**G**) brown adipocytes (BAT) in mice. Specimens were photographed using light microscopy. (Hematoxylin and eosin stain, magnification: 200×; scale bar, 40 or 80 μm).

Figure 7 shows the results of the inspections of different tissues (liver, muscle, heart, kidney, and fat pad) for potential pathological changes with programmed training and resveratrol supplementation. The arrangement of sinusoid and hepatic cords in the liver showed no changes after the indicated treatments. In addition, Zenker’s degeneration and hyperplasia were not observed in the skeletal muscles or cardiomyocytes, and the structure of the renal tubules and glomeruli did not differ between the treatment groups. The white adipose tissue (WAT) was composed of adipocytes, which are very large cells that have small, uniform nuclei and are usually located near the plasma membrane. Most of the cytoplasm in mice is occupied by a large lipid drop, which was found to be empty in most of the histological slides because fat was removed during the histological process. The cellular size of brown adipocytes (BAT) is usually smaller than that of white adipocytes. These morphologic differences may be because the cytoplasm is distributed with small, fatty droplets. Moreover, the nucleus is circular and located at the center of the cell.

### 3.9. The Effects of Climb Training and Resveratrol on Muscle Types and Morphology

**Figure 8:**
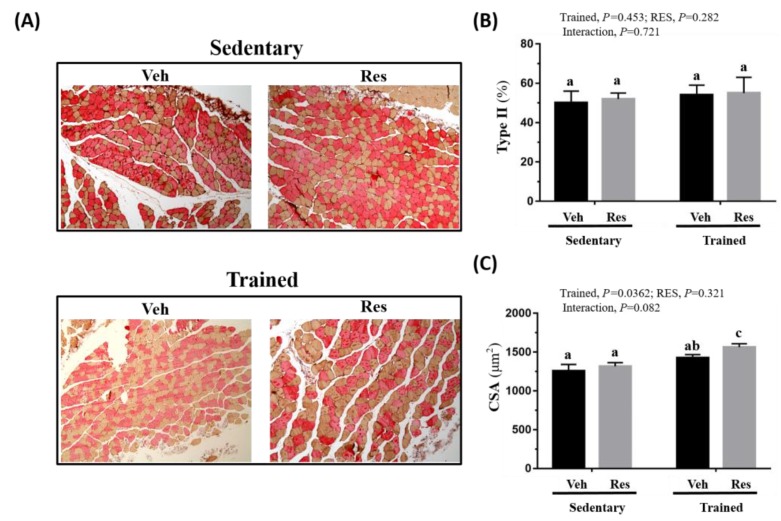
Effect of climb training (Trained) and/or resveratrol (RES) supplementation on the muscle of thigh with IHC staining (**A**), muscular type proportions (**B**), and cross section area (CSA) (**C**). Specimens were photographed under a light microscope. (Hematoxylin and eosin stain, magnification: 200×; scale bar, 40 μm). Bars with different superscript letters (a, b, c) are significantly different at *P* < 0.05. The abbreviations Veh and RES represented the vehicle and resveratrol supplement, respectively.

Figure 8A shows the results of further analysis of the type I and type II fiber proportions and cross section area (CSA) of the thigh muscle that was conducted to verify the effect of resistance exercise and RES supplementation. Type I and type IIa were reddish in color, while type II was brownish in color. The proportions of muscular fiber in the different groups did not differ significantly after resistance training and/or RES treatment and the cross-section area of the indicated groups showed a significant training effect, as seen in Figure 8B,C. The CSA of the Trained + RES group was significantly higher than that of other groups.

## 4. Discussion

In this study, RES and programmed resistance exercise elevated the aerobic endurance effectively over the 4-week intervention, possibly due to the modulation of lactate metabolite and glycogen content, which is a finding consistent with previous studies [17,26]. The resistance training was shown to improve strength and muscular hypertrophy, while the RES, in combination with the 4-week resistance training program, demonstrated a significantly synergistic increase not only in terms of anaerobic performance and endurance but also of the exercise-induced lactate production for better physiological adaption. The treatments of RES, programmed resistance training, and combination also elucidated the safety with growth curve, biochemistries, and histopathology.

Although aerobic capacity may be improved using traditional, low-intensity endurance training, or high-intensity resistance, circuit-based training also demonstrated a significantly positive effect on aerobic performance [27]. The data collected for this study, shown in Figure 5, demonstrated that a combination of RES supplementation and resistance training could significantly improve aerobic performance with respective main effects via a treadmill platform. This finding is consistent with previous studies regarding the effects of resistance exercise on cardiopulmonary fitness and the RES anti-fatigue effect [17], but no interaction effect was observed in the present result. In addition, the climb training with incremental load has been widely applied to animal studies as the resistance exercise model [28]. As shown in Figure 1B, the forelimb and pelvic limb are required to climb firmly and fight against the resistance generated by the screw washer. The elevation of grip strength was highly associated with both the resistance exercise [29] and the RES-improved ultrastructure of the myofibrils via the activating AMPK/sirt1 pathway [30]. However, this study found that RES in combination with resistance exercise demonstrated a significantly synergistic effect with the interaction effect in terms of both absolute and relative grip strength, as seen in Figure 3A,B.

Power (explosiveness) and speed endurance are important factors in the success of athletes in various sports. Resistance training has the potential to improve both strength and power as a result of hypertrophy and neural adaptations in beginner athletes, but not in advanced athletes [31]. Therefore, increasing strength increases power performance, while power training may assist in the development of endurance performance [32]. Thus, the time duration of each climb in the indicated groups permitted an assessment of muscular power capacity in a short time period due to rodent aquaphobia. In Figure 4A, RES and resistance also showed their respective main effects, while their combined effects demonstrated the significant interaction effect of the higher synergistic effect on power performance. The model of resistance exercise in this study models the concept of higher-intensity interval training for the improvement of cardiopulmonary and muscular endurance performance with similar aerobic and anaerobic adaptations [33]. Therefore, the aerobic performance, shown in Figure 5, and anaerobic endurance, shown in Figure 4B, may be explained as the main effect of training and possible interaction effect, respectively, in this study.

Different muscle groups may be involved in specific physical movements, as resistance exercise is known to induce muscle hypertrophy. The muscle groups that were involved in the exercise regimen in this study included the quadriceps, biceps femoris, gluteus maximus, and gastrocnemius muscles for functional movement, like climbing stairs or squats. The ladder-climbing animal model simulates the muscle hypertrophy in leg muscle groups caused by resistance exercise, especially with programmed weight loads. In previous studies, progressive resistance exercise was shown to induce muscle hypertrophy in the cross-section area [28] and tissue weight [34] in the rodent animal model. The results of this study found a significant difference in terms of the main effect of training, which means that resistance training contributed to hypertrophy in the muscle of thigh, as shown in Table 3. Moreover, analysis of the immunohistochemical staining and cross section area (CSA) on the muscle of thigh showed a significant increase in the CSA with the main effect of training but without an interaction effect, as shown in Figure 8. This result may directly explain the possible functional increase on grip strength and anaerobic performance.

The effects of the interventions were evaluated using the exercise-associated metabolites and energy content to infer physiological adaption. Lactate, which relates positively to exercise duration and intensity, releases hydrogen ions, which have potentially deleterious effects on metabolism and on the release of calcium during muscular contractions [35], eventually contributing to the sensation of fatigue. Previous reports have found that HIIT training improves insulin sensitivity and glycogen content [36]. In addition, resveratrol is known to improve insulin sensitivity and to mimic the effects of calorie restriction in terms of reducing blood glucose levels, insulin levels, and insulin like growth factor-1 (IGF-1) via the activation of the longevity gene SirT1 [37]. Furthermore, the resveratrol affects upregulated mitochondrial function and aerobic capacity through the activation of muscle SirT1 and PGC alpha [38]. Therefore, the aerobic capacity during the treadmill test may be elucidated by the possible effects of resveratrol and training. On the other hand, although the administrative dose of RES 25 mg/kg did not elevate the glycogen content, which was consistent with our previous report [17], the glycogen content of training combined with RES was significantly higher than in the other groups with the main effects of training in this study, as seen in Figure 6. The content and availability of glycogen have a critical effect on the anaerobic capacity of type II muscle fiber, as shown in the climbing test in Figure 4. However, while the proportions of muscular type were not significantly altered in the current intervention, the training combined with RES supplement was significantly higher than other indicated groups with the main effects of training as shown in Figure 8B,C.

This study presumed that the synergistic effects of resistance exercise combined with resveratrol supplement could not be observed in terms of aerobic performance, muscular fiber compositions, and muscular hypertrophy. Thus the two issues of resveratrol bioavailability and resistance exercise protocol should be examined further in order to understand their combinational benefits and mechanisms. The oral bioavailability of resveratrol in humans is quite limited, but may be increased significantly without any treatment-related adverse effects through the use of a liquid micellar formulation [39]. The dose in current animal studies could be further converted to human dose according to the conversion factor of 12.3-fold by body surface area difference, suggested by US Food and Drug Administration [40]. Before the clinical trial, we will test the resveratrol pharmacokinetics for bioavailability with different formulation. The preferred subject would be a non-athlete to avoid the training effects, and the programmed resistance training would be intervened with optimized resveratrol formulation supplement. In addition, the body composition, muscular strength, power, aerobic, and anaerobic capacities would be assessed for functional and physiological validation.

## 5. Conclusions

Exercise duration and loading intensity may be elevated further for better physiological adaptions. Therefore, there may be value in further studying the potential molecular mechanisms that underlie these combinational effects. In terms of practical applications, people have limited time for exercise. Therefore, effective and efficient methods of exercise are particularly appropriate for promoting public fitness and health. This study supports the hypothesis that resistance exercise in combination with resveratrol supplementation effectively induces muscular hypertrophy, physiological adaption, aerobic, and anaerobic performance.

## Figures and Tables

**Table 1:** Effects of climb training (Trained) and/or resveratrol (RES) on fatigue-related biochemical assessments of serum after acute exercise.

					*F* Values for Two-Way ANOVA		
Parameter	Sed + Vehicle	Sed + RES	Train + Vehicle	Train + RES	Main Effect of RES	Main Effect of Climb	Interaction (RES × Climb)
**GLU (mg/dL)**	103 ± 5	116 ± 11	124 ± 9	123 ± 11	0.45	2.16	0.5
**LACT (mmol/L)**	4.9 ± 0.2 ^b^	4.5 ± 0.2 ^b^	4.7 ± 0.2 ^b^	3.5 ± 0.2 ^a^	19.84 *	9.79 *	4.77 *
**BUN (mg/dL)**	25.8 ± 0.8	23.9 ± 0.6	23.7 ± 1	23.9 ± 1	0.95	1.44	1.5
**CK (U/L)**	395 ± 65	349 ± 62	459 ± 73	364 ± 52	1.03	0.47	0.1
**NH_3_ (umol/L)**	97 ± 46 ^a,b^	90 ± 78 ^a^	106 ± 28 ^b^	85 ± 59 ^a^	8.02 *	0.16	2.24

Values are the mean ± SEM for *n* = 10 mice in each group. Values in the same line with different superscripts letters (a, b) differ significantly; *: *P* < 0.05 by two-way analysis of variance (ANOVA). GLU: glucose; LACT: lactate; BUN: blood urine nitrogen; CK: creatine kinase; NH_3_: ammonia.

**Table 2:** Effects of climb training (Trained) and/or resveratrol (RES) on biochemical assessments of serum at the end of the experiment.

					*F* Values for Two-Way ANOVA		
Parameter	Sed + Vehicle	Sed + RES	Trained + Vehicle	Trained + RES	Main Effect of RES	Main Effect of Climb	Interaction (RES × Climb)
**AST (U/L)**	129 ± 12	149 ± 22	116 ± 8	174 ± 37	3	0.07	0.68
**ALT (U/L)**	44 ± 3 ^a^	78 ± 12 ^b^	50 ± 5 ^a,b^	77 ± 17 ^b^	7.9 *	0.06	0.1
**TG (mg/dL)**	114 ± 5	118 ± 9	124 ± 12	125 ± 9	0.06	0.81	0.02
**CK (U/L)**	400 ± 48	384 ± 83	360 ± 36	349 ± 61	0.05	0.4	0
**LDH (U/L)**	565 ± 46	672 ± 78	547 ± 28	634 ± 59	3.07	0.26	0.03
**BUN (mg/dL)**	24.5 ± 0.6	25.4 ± 1.1	25.8 ± 0.6	26.5 ± 1.3	0.64	1.5	0.01
**CREA (mg/dL)**	0.4 ± 0.01	0.4 ± 0.01	0.4 ± 0.01	0.4 ± 0.01	0	0	2
**UA (mg/dL)**	1.83 ± 0.14 ^a^	2.91 ± 0.31 ^b^	3.05 ± 0.39 ^b,c^	3.90 ± 0.40 ^c^	8.79 *	11.53 *	0.12
**ALB (g/dL)**	2.7 ± 0.1	2.7 ± 0.1	2.8 ± 0.1	2.8 ± 0.1	0.66	0.27	0.14
**TP (g/dL)**	5.4 ± 0.1	5.4 ± 0.1	5.5 ± 0.1	5.4 ± 0.1	0.67	0.05	0.12
**GLU**	148 ± 8	142 ± 10	157 ± 10	143 ± 6	1.34	0.28	0.25

Values are the mean ± SEM for *n* = 10 mice in each group. Values in the same line with different superscripts letters (a, b, c) differ significantly; *: *P* < 0.05 by two-way analysis of variance (ANOVA). Sed and Trained refer to the sedentary and climbing intervention, respectively. AST, aspartate aminotransferase; ALT, alanine aminotransferase; ALB, albumin; LDH, lactate dehydrogenase; TP, total protein; BUN, blood urea nitrogen; CREA, creatine; UA, uric acid; TG, triacylglycerol; GLU, glucose.

**Table 3:** General characteristics of the experimental groups.

					*F* Values for Two-Way ANOVA		
Characteristic	Sed + Vehicle	Sed + RES	Trained + Vehicle	Trained + RES	Main Effect of RES	Main Effect of Climb	Interaction (RES × Climb)
**Initial BW (g)**	31.0 ± 0.3	30.4 ± 0.3	30.3 ± 0.2	30.7 ± 0.4	---	---	---
**Final BW (g)**	38.5 ± 0.4	37.7 ± 0.6	38.4 ± 0.6	38.5 ± 0.6	0.16	0.21	0.31
**Food intake (g/day)**	7.28 ± 0.2 ^a^	7.84 ± 0.2 ^c^	7.6 ± 0.2 ^b^	7.2 ± 0.2 ^a^	0.46	5.6^*^	44.69 *
**Water intake (mL/day)**	9.5 ± 0.3 ^a^	10.14 ± 0.2 ^b^	10.73 ± 0.2 ^c^	10.64 ± 0.2 ^c^	7.9 *	77.81 *	14.27 *
**Liver (g)**	2.04 ± 0.17	2.04 ± 0.16	2.15 ± 0.12	2.10 ± 0.16	0.1	1.31	0.17
**Muscle (g)**	0.38 ± 0.05	0.36 ± 0.06	0.38 ± 0.05	0.39 ± 0.07	1.25	0.77	0.52
**MT (g)**	0.46 ± 0.02 ^a^	0.48 ± 0.02 ^a,b^	0.51 ± 0.02 ^b,c^	0.53 ± 0.02 ^c^	0.822	10.1 *	0.01
**Heart (g)**	0.17 ± 0.04	0.18 ± 0.04	0.18 ± 0.05	0.18 ± 0.04	0.56	0.2	0.2
**Lung (g)**	0.26 ± 0.07	0.23 ± 0.07	0.24 ± 0.05	0.23 ± 0.06	1.25	0.77	0.52
**Kidney (g)**	0.68 ± 0.06	0.67 ± 0.07	0.68 ± 0.07	0.68 ± 0.08	0.46	0.03	0.03
**EFP (g)**	0.39 ± 0.11	0.35 ± 0.14	0.39 ± 0.12	0.34 ± 0.12	0.98	0	0.01
**BAT (g)**	0.11 ± 0.04	0.10 ± 0.06	0.10 ± 0.04	0.16 ± 0.13	0.48	1	1.22

Values in the same line with different superscripts letters (a, b, c) differ significantly; *: *P* < 0.05 by two-way analysis of variance (ANOVA). Sed and Trained refer to the sedentary and climbing intervention, respectively. Muscle: gastrocnemius and soleus muscles; MT: muscle of thigh; EFP: epididymal fat pad; BAT: brown adipocyte tissue; RES: resveratrol.

## References

[1] Kulkarni S.S., Cantó C. (2015). The molecular targets of resveratrol. Biochim. Biophys. Acta.

[2] Sales J.M., Resurreccion A.V. (2014). Resveratrol in peanuts. Crit. Rev. Food. Sci. Nutr..

[3] Romero-Pérez A.I., Ibern-Gómez M., Lamuela-Raventós R.M., de La Torre-Boronat M.C. (1999). Piceid, the major resveratrol derivative in grape juices. J. Agric. Food Chem..

[4] Biagi M., Bertelli A.A. (2015). Wine, alcohol and pills: What future for the French paradox?. Life Sci..

[5] Zheng X., Jia B., Song X., Kong Q.Y., Wu M.L., Qiu Z.W., Li H., Liu J. (2018). Preventive Potential of Resveratrol in Carcinogen-Induced Rat Thyroid Tumorigenesis. Nutrients.

[6] Marques B.C.A.A., Trindade M., Aquino J.C.F., Cunha A.R., Gismondi R.O., Neves M.F., Oigman W. (2018). Beneficial effects of acute trans-resveratrol supplementation in treated hypertensive patients with endothelial dysfunction. Clin. Exp. Hypertens..

[7] Chen S., Zhao Z., Ke L., Li Z., Li W., Zhang Z., Zhou Y., Feng X., Zhu W. (2018). Resveratrol improves glucose uptake in insulin-resistant adipocytes via Sirt1. J. Nutr. Biochem..

[8] Peñalver P., Belmonte-Reche E., Adán N., Caro M., Mateos-Martín M.L., Delgado M., González-Rey E., Morales J.C. (2018). Alkylated resveratrol prodrugs and metabolites as potential therapeutics for neurodegenerative diseases. Eur. J. Med. Chem..

[9] Li Y.R., Li S., Lin C.C. (2018). Effect of resveratrol and pterostilbene on aging and longevity. Biofactors.

[10] Schoenfeld B.J. (2010). The mechanisms of muscle hypertrophy and their application to resistance training. J. Strength Cond. Res..

[11] Flack K.D., Davy K.P., Hulver M.W., Winett R.A., Frisard M.I., Davy B.M. (2010). Aging, resistance training, and diabetes prevention. J. Aging Res..

[12] Siparsky P.N., Kirkendall D.T., Garrett W.E. (2014). Muscle Changes in Aging: Understanding Sarcopenia. Sports Health.

[13] Barcelos C., Damas F., Nóbrega S.R., Ugrinowitsch C., Lixandrão M.E., Marcelino Eder Dos Santos L., Libardi C.A. (2018). High-frequency resistance training does not promote greater muscular adaptations compared to low frequencies in young untrained men. Eur. J. Sport Sci..

[14] Westcott W.L. (2012). Resistance training is medicine: Effects of strength training on health. Curr. Sports Med. Rep..

[15] Buckner S.L., Jessee M.B., Dankel S.J., Mattocks K.T., Abe T., Loenneke J.P. (2018). Resistance exercise and sports performance: The minority report. Med. Hypotheses.

[16] Lloyd R.S., Faigenbaum A.D., Stone M.H., Oliver J.L., Jeffreys I., Moody J.A., Brewer C., Pierce K.C., McCambridge T.M., Howard R. (2014). Position statement on youth resistance training: The 2014 International Consensus. Br. J. Sports Med..

[17] Wu R.E., Huang W.C., Liao C.C., Chang Y.K., Kan N.W., Huang C.C. (2013). Resveratrol protects against physical fatigue and improves exercise performance in mice. Molecules.

[18] Alway S.E., McCrory J.L., Kearcher K., Vickers A., Frear B., Gilleland D.L., Bonner D.E., Thomas J.M., Donley D.A., Lively M.W. (2017). Resveratrol enhances exercise-Induced cellular and functional adaptations of skeletal muscle in older men and women. J. Gerontol. A Biol. Sci. Med. Sci..

[19] Gliemann L., Schmidt J.F., Olesen J., Biensø R.S., Peronard S.L., Grandjean S.U., Mortensen S.P., Nyberg M., Bangsbo J., Pilegaard H. (2013). Resveratrol blunts the positive effects of exercise training on cardiovascular health in aged men. J. Physiol..

[20] Hornberger T.A., Farrar R.P. (2004). Physiological hypertrophy of the FHL muscle following 8 weeks of progressive resistance exercise in the rat. Can. J. Appl. Physiol..

[21] Conover C.A., Bale L.K., Nair K.S. (2016). Comparative gene expression and phenotype analyses of skeletal muscle from aged wild-type and PAPP-A-deficient Mice. Exp. Gerontol..

[22] Hsu Y.J., Huang W.C., Chiu C.C., Liu Y.L., Chiu W.C., Chiu C.H., Chiu Y.S., Huang C.C. (2016). Capsaicin supplementation reduces physical fatigue and improves exercise performance in mice. Nutrients.

[23] Huang W.C., Huang C.C., Chuang H.L., Chen W.C., Hsu M.C. (2017). Cornu cervi pantotrichum supplementation improves physiological adaptions during intensive endurance training. J. Vet. Med. Sci..

[24] Hsiao C.Y., Chen Y.M., Hsu Y.J., Huang C.C., Sung H.C., Chen S.S. (2017). Supplementation with Hualian No. 4 wild bitter gourd (Momordica charantia Linn. var. abbreviata ser.) extract increases anti-fatigue activities and enhances exercise performance in mice. J. Vet. Med. Sci..

[25] Musa T.H., Li W., Xiaoshan L., Guo Y., Wenjuan Y., Xuan Y., YuePu P., Pingmin W. (2018). Association of normative values of grip strength with anthropometric variables among students, in Jiangsu Province. Homo.

[26] Prestes J., Leite R.D., Pereira G.B., Shiguemoto G.E., Bernardes C.F., Asano R.Y., Sales M.M., Bartholomeu Neto J., Perez S.E. (2012). Resistance training and glycogen content in ovariectomized rats. Int. J. Sports Med..

[27] Ramos-Campo D.J., Martínez-Guardado I., Olcina G., Marín-Pagán C., Martínez-Noguera F.J., Carlos-Vivas J., Alcaraz P.E., Rubio J.Á. (2018). Effect of high-intensity resistance circuit-based training in hypoxia on aerobic performance and repeat sprint ability. Scand. J. Med. Sci. Sports.

[28] Hellyer N.J., Nokleby J.J., Thicke B.M., Zhan W.Z., Sieck G.C., Mantilla C.B. (2012). Reduced ribosomal protein s6 phosphorylation after progressive resistance exercise in growing adolescent rats. J. Strength Cond. Res..

[29] Olvera-Soto M.G., Valdez-Ortiz R., López Alvarenga J.C., Espinosa-Cuevas Mde L. (2016). Effect of resistance exercises on the indicators of muscle reserves and handgrip strength in adult patients on hemodialysis. J. Ren. Nutr..

[30] Liao Z.Y., Zhao K.X., Xiao Q. (2017). Effect of resveratrol on forelimb grip strength and myofibril structure in aged rats. Nan Fang Yi Ke Da Xue Xue Bao.

[31] Wilson G.J., Newton R.U., Murphy A.J., Humphries B.J. (1993). The optimal training load for the development of dynamic athletic performance. Med. Sci. Sports Exerc..

[32] Paavolainen L., Häkkinen K., Hämäläinen I., Nummela A., Rusko H. (1999). Explosive-strength training improves 5-km running time by improving running economy and muscle power. J. Appl. Physiol..

[33] Buckley S., Knapp K., Lackie A., Lewry C., Horvey K., Benko C., Trinh J., Butcher S. (2015). Multimodal high-intensity interval training increases muscle function and metabolic performance in females. Appl. Physiol. Nutr. Metab..

[34] Jung S., Ahn N., Kim S., Byun J., Joo Y., Kim S., Jung Y., Park S., Hwang I., Kim K. (2015). The effect of ladder-climbing exercise on atrophy/hypertrophy-related myokine expression in middle-aged male Wistar rats. J. Physiol. Sci..

[35] Cairns S.P. (2006). Lactic acid and exercise performance: Culprit or friend?. Sports Med..

[36] Søgaard D., Lund M.T., Scheuer C.M., Dehlbaek M.S., Dideriksen S.G., Abildskov C.V., Christensen K.K., Dohlmann T.L., Larsen S., Vigelsø A.H., Dela F. (2018). High-intensity interval training improves insulin sensitivity in older individuals. Acta Physiol..

[37] Lekli I., Ray D., Das D.K. (2010). Longevity nutrients resveratrol, wines and grapes. Genes Nutr..

[38] Momken I., Stevens L., Bergouignan A., Desplanches D., Rudwill F., Chery I., Zahariev A., Zahn S., Stein T.P., Sebedio J.L. (2011). Resveratrol prevents the wasting disorders of mechanical unloading by acting as a physical exercise mimetic in the rat. FASEB J..

[39] Calvo-Castro L.A., Schiborr C., David F., Ehrt H., Voggel J., Sus N., Behnam D., Bosy-Westphal A., Frank J. (2018). The Oral bioavailability of trans-resveratrol from a grapevine-shoot extract in healthy humans is significantly increased by micellar solubilization. Mol. Nutr. Food Res..

[40] Nair A.B., Jacob S. (2016). A simple practice guide for dose conversion between animals and human. J. Basic Clin. Pharm..

